# Leaf Area Index Estimation Using Chinese GF-1 Wide Field View Data in an Agriculture Region

**DOI:** 10.3390/s17071593

**Published:** 2017-07-08

**Authors:** Xiangqin Wei, Xingfa Gu, Qingyan Meng, Tao Yu, Xiang Zhou, Zheng Wei, Kun Jia, Chunmei Wang

**Affiliations:** 1 Institute of Remote Sensing and Digital Earth, Chinese Academy of Sciences, Beijing 100101, China; weixq@radi.ac.cn (X.W.); mengqy@radi.ac.cn (Q.M.); yutao@radi.ac.cn (T.Y.); zhouxiang@radi.ac.cn (X.Z.); wangcm@radi.ac.cn (C.W.); 2 University of Chinese Academy of Sciences, Beijing 100049, China; 3 Application Technology Center of China High-Resolution Earth Observation System, Beijing 100101, China; weizheng@irsa.ac.cn; 4 State Key Laboratory of Remote Sensing Science, Faculty of Geographical Science, Beijing Normal University, Beijing 100875, China

**Keywords:** leaf area index, radiative transfer model, neural networks, GF-1 satellite, wide field view

## Abstract

Leaf area index (LAI) is an important vegetation parameter that characterizes leaf density and canopy structure, and plays an important role in global change study, land surface process simulation and agriculture monitoring. The wide field view (WFV) sensor on board the Chinese GF-1 satellite can acquire multi-spectral data with decametric spatial resolution, high temporal resolution and wide coverage, which are valuable data sources for dynamic monitoring of LAI. Therefore, an automatic LAI estimation algorithm for GF-1 WFV data was developed based on the radiative transfer model and LAI estimation accuracy of the developed algorithm was assessed in an agriculture region with maize as the dominated crop type. The radiative transfer model was firstly used to simulate the physical relationship between canopy reflectance and LAI under different soil and vegetation conditions, and then the training sample dataset was formed. Then, neural networks (NNs) were used to develop the LAI estimation algorithm using the training sample dataset. Green, red and near-infrared band reflectances of GF-1 WFV data were used as the input variables of the NNs, as well as the corresponding LAI was the output variable. The validation results using field LAI measurements in the agriculture region indicated that the LAI estimation algorithm could achieve satisfactory results (such as R^2^ = 0.818, RMSE = 0.50). In addition, the developed LAI estimation algorithm had potential to operationally generate LAI datasets using GF-1 WFV land surface reflectance data, which could provide high spatial and temporal resolution LAI data for agriculture, ecosystem and environmental management researches.

## 1. Introduction

Vegetation is the basic component of the terrestrial ecosystem and plays an important role in energy exchange, carbon cycling and hydrological cycling process on the earth surface [1,2,3]. Therefore, timely and accurate land surface vegetation information is of great significance for earth system science, ecological environment assessment and climate change related studies [4]. Leaf area index (LAI), generally defined as one half of the total green leaf area per unit of horizontal ground surface area [5], is an important parameter for characterizing land surface vegetation conditions [6,7,8]. LAI is an essential parameter that characterizes the density of leaves and canopy structure, which can reflect the vegetation’s ability for biophysical processes such as photosynthesis, respiration and transpiration, and is also an important input variable for the carbon cycle models, crop growth models and water cycle models [9,10,11,12,13]. Therefore, accurate estimation of LAI on regional and global scales is of great significance for ecosystem modelling, biogeochemical cycle modelling, agriculture monitoring and related applications.

Remote sensing provides the only effective means to estimate LAI at the regional and global scales for its ability of continuous observation and to provide broad and impartial earth observation data [7,8,14,15]. Currently, several global LAI products have been generated using medium to low spatial resolution satellite data, such as the MODIS LAI products [6], GEOV1 LAI Products [7], MERIS LAI products [16], GLASS LAI products [17] and GLOBCARBON LAI products [18]. However, the currently existed LAI products generally have low spatial resolutions at the kilometric level, whereas decametric spatial resolution LAI data will be better suited for applications related to agriculture monitoring, as compared to kilometric resolution LAI data, which are usually larger than the typical scales of most croplands. The wide field view (WFV) sensor on board GF-1, the first satellite of the China High-resolution Earth Observation System, can acquire multi-spectral data with high spatial and temporal resolutions [19], which are valuable data sources for dynamic LAI monitoring at regional scale. Therefore, exploring the application potential of GF-1 WFV data on land surface LAI estimation and developing the specific LAI estimation algorithm for GF-1 WFV data are urgently needed.

The key of LAI estimation using remote sensing data is how to establish the relationship between LAI and land surface reflectance data according to the radiative transfer process of photon in the vegetation canopy and its spectral response characteristics [20,21,22,23]. Many algorithms have been developed to retrieve LAI using satellite remote sensing data and generally two types of algorithms can be distinguished including empirical methods and physical model based methods [6,24,25,26,27]. The empirical LAI estimation methods are based on the statistical relationships between LAI and vegetation indices (VIs), which are calibrated using field LAI measurements and remote sensing data or simulated data from canopy radiative transfer models [28,29]. The empirical methods simplify the complex radiative transfer process in the canopy, which are simple and can obtain satisfactory LAI estimation accuracy in small regions. Furthermore, when using VIs as independent variables, the VIs can highlight vegetation information and weaken the influences of canopy shadows, soil backgrounds, atmospheric conditions and angle effects [29]. However, the empirical methods only use the VIs calculated from several band reflectances, which cannot make full use of the multi-band spectral information of remote sensing data. Furthermore, the reduction of the VIs from multi-band reflectances to an index also reduces the LAI estimation constraints and increases the uncertainty of LAI estimation results, resulting in the empirical relationship between LAI and VIs changing with sensor types, vegetation types, time and geographical areas. Therefore, the application of empirical LAI estimation methods to a large scale is very difficult, due to the complexity of land surface.

The physical model based methods are mainly based on the simulation of radiative transfer in vegetation canopy and establishing the physical relationship between canopy reflectances and LAI, and then the canopy radiative transfer models are inversed [30,31]. The direct inversion of radiative transfer models is very difficult due to the complexity of the models, and iterative optimization (OPT) method [32], lookup table (LUT) method [6] and machine learning methods [26] are usually used for indirect inversion of physical models to achieve LAI estimation. The OPT method is based on the iterative minimization of a cost function which requires hundreds of runs of the canopy radiative transfer model for each pixel and therefore computationally too demanding. For practical applications, LUT method and machine learning methods are popular LAI estimation methods, which are based on the database simulated by physical models. The LUT method is conceptually the simplest technique by finding the solution for a given set of reflectance measurements, which consisted of selecting the closest cases in the database according to a cost function, and then based on extracting the corresponding set of LAI [6,33]. However, the LUT method usually requires a fixed number of input variables, unless there are very large lookup tables, which are difficult to manipulate. Machine learning methods can efficiently and accurately approximate the complex nonlinear functions, and train the algorithm parameters through training samples to realize the efficient and accurate estimation of LAI using remote sensing data [26,34]. Commonly used machine learning methods mainly include neural networks (NNs), as well as support vector machines and decision trees [35]. NNs trained over radiative transfer model simulations have been applied, with success, to estimate LAI from several sensors’ data, leading to several operational LAI production algorithms, such as the CYCLOPES and MERIS LAI products [26,36]. Therefore, based on the reality of work in the field of LAI estimation using remote sensing data, the NNs inversion of physical models is a potential and reasonable choice for LAI estimation from GF-1WFV data. 

Therefore, the objective of this study is to develop a general LAI estimation algorithm for GF-1 WFV reflectance data under various land surface conditions using NNs trained over radiative transfer model simulations. Meanwhile, the field LAI measurements in an agriculture region with maize as the dominated crop type are used to assess the performance of GF-1 WFV data on LAI estimation using the proposed algorithm.

## 2. Study Area

**Figure 1:**
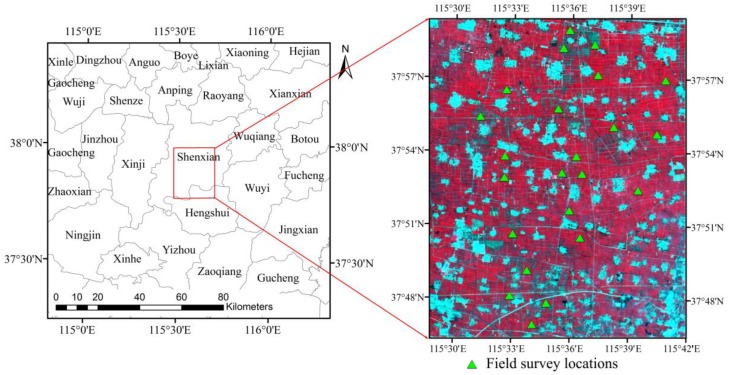
Square region in the left image shows the geo-location of the Shenzhou study area in Hebei Province, and the right image is the GF-1 WFV data acquired on 15 August 2014. Generally, the red patches are farmland in the right image and the blue patches are non-vegetated regions, such as residential areas and roads. The green triangles on the right image indicate the field survey locations.

The Shenzhou county of China (Figure 1) is selected as the study area to investigate the performance of LAI estimation algorithm for GF-1 WFV data. The study area is located on the North China Plain (centred at 115°35′ E, 37°53′ N) covering approximately 20 km × 25 km. The study area belongs to the temperate climate zone and is a typical upland field agriculture area in the North China Plain. The annual average temperature is approximately 13.4 °C, and the annual average precipitation is approximately 486 mm, which is mainly concentrated in July and August. It is relatively flat farmland with an average altitude of about 20 m above the sea level, so that uncertainties of LAI estimation caused by topographical facts will be reduced to a minimum. The main autumn grain crops planted in the study area are maize. Maize season begins in mid-June and harvests in early October. Though the study area is not big, it has the representative characteristics of crop type distribution in the North China Plain.

## 3. Data and Pre-Processes

### 3.1. Field LAI Measurements

In order to validate the LAI estimation accuracy using GF-1 WFV data, the field campaigns were completed covering the main growing seasons of maize, and the specific field observation dates were 27 June, 21 July, 14 August and 5 September 2014. Twenty-three representative square sample sites (30 m × 30 m) within the study area (Figure 1) were selected based on the crop growth conditions and the high spatial resolution remote sensing data. The sample sites were located in relatively homogeneous regions with approximately 50 m around the sample sites having similar crop growth conditions, thus the uncertainty of LAI estimation caused by the pixel matching error between GF-1 WFV data and the sample site would be minimized. The center of each sample site was determined using the handheld global positioning system (GPS) receiver which had a positioning accuracy of approximately ±3 m. LAI was measured using an LAI-2000 plant canopy analyzer (Li-Cor, Inc., Lincoln, NE, USA), and there were three measuring plots in each sample site and the average LAI value of the three LAI measurements was regarded as the LAI of the sample site. The number of LAI measurement sites on 27 June was four because maize in this period was very small, and on 21 July, 14 August and 5 September were all twenty-three. These field LAI measurements were used to validate the LAI estimates using the GF-1 WFV data. 

### 3.2. GF-1 WFV Data and Pre-Processes

In order to validate the proposed LAI estimation algorithm, GF-1 WFV data synchronized with the field LAI measurements were required. The spatial resolution of GF-1 WFV data was 16 m and the temporal resolution was four days for the four WFV sensors combined. According to the field survey dates and the image quality of GF-1 WFV data, the high-quality GF-1 WFV data close to the field survey dates were selected as much as possible, and finally 5 GF-1 WFV data were collected (Table 1). The GF-1 WFV data acquired on 24 June, 18 July and 15 August 2014 were close to the corresponding field survey dates (3 days or less), which could be approximated that LAI in this close time did not change and the field LAI measurements could be directly used to validate the LAI estimates using GF-1 WFV data. However, corresponding to the field LAI measurements on 5 September 2014, there were no suitable GF-1 WFV data close to the field survey date due to the effect of clouds, thus the GF-1 WFV data acquired on 24 August and 18 September 2014 were selected to estimate LAI, which would be interpolated to estimate LAI on 5 September using the spline interpolation method and then the LAI estimation accuracy would be validated using the filed LAI measurements on 5 September.

The pre-process of GF-1 WFV data contained radiance calibration, atmospheric correction and geometric correction. The radiance calibration was converting the DN values of the raw data to radiances using the following equation:

$$L_{e}\;=\;\mathrm{Gain}\;\times \mathrm{DN}\;+\;\mathrm{Offset}$$

where L_e_ was the radiance, and Gain and Offset were calibration coefficients obtained from the China Centre for Resources Satellite Data and Application, which were listed in Table 2.

The Fast Line-of-sight Atmospheric Analysis of Spectral Hypercubes (FLAASH) model was used to atmospheric correction of GF-1 WFV data [37]. The input parameters for FLAASH model were determined based on the imaging time and imaging parameters of each GF-1 WFV data. After the atmospheric correction, the GF-1 WFV land surface reflectance data were obtained and the reflectances of green, red and near-infrared (NIR) bands would be used to estimate LAI. The geometric correction of GF-1 WFV data was conducted using two-order polynomial transformation method with bilinear interpolation resampling. High quality Landsat-8 Operational Land Imager data [38] were selected as the base map, and ground control points were selected from the images manually. The resulted geometric co-registration error for each GF-1 WFV data was less than one pixel of the Landsat data (30 m). Finally, the subset images of the atmospherically and geometrically corrected GF-1 WFV data covered the sample sites were extracted to further investigate the performance of the LAI estimation algorithm for GF-1 WFV data (Figure 1).

## 4. Methods

**Figure 2:**
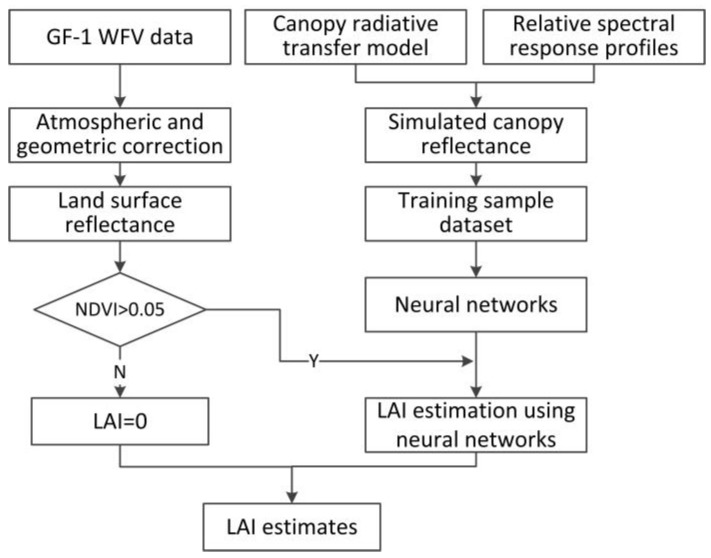
Flowchart of the leaf area index (LAI) estimation algorithm for GF-1 WFV data.

A flowchart of the LAI estimation algorithm development for GF-1 WFV data was presented in Figure 2. The training sample dataset was firstly generated using the spectral reflectance simulations based on the radiative transfer model and the relative spectral response profiles of GF-1 WFV sensors. Then, the NNs were trained over the training sample dataset to develop the LAI estimation algorithm for GF-1 WFV data. Finally, LAI could be estimated using the pre-processed GF-1 WFV land surface reflectance data using the trained NNs or set to zero when the pixel was non-vegetation which was judged by the normalized difference vegetation index (NDVI) value. 

### 4.1. Generating LAI Training Sample Dataset from Radiative Transfer Model Simulations

The canopy radiative transfer model quantitatively described the physical relationship between LAI and canopy spectral reflectances. The widely used coupled PROSPECT and SAIL (PROSAIL) model, which was easy to use, had general robustness and also had consistent performance in validation practices [22,39,40] was selected to simulate the satellite observations of canopy reflectance based on the relative spectral response profiles of the GF-1 WFV sensors. The PROSPECT model was a plate model based radiative transfer model, which simulated the hemispherical reflectance and transmittance of leaves from spectral wavelength from 0.4 to 2.5 μm based on the leaf biochemical and biophysical parameters [41,42]. The input parameters for PROSPECT model included leaf chlorophyll a + b concentration (C_ab_), water content (C_w_), dry matter content (C_m_), brown pigment content (C_brown_), carotenoid content (C_ar_) and leaf structure parameter (N), and the output parameters were hemispherical reflectance and transmittance of leaves, which were also the input parameters for SAIL model. The SAIL model with hot-spot correction, which assumed the canopy as a turbid medium, was selected in this study [22,26]. The canopy structure in the SAIL model was characterized by LAI, the average leaf angle inclination (ALA) assuming an ellipsoidal distribution and the hot-spot parameter. 

**Figure 3:**
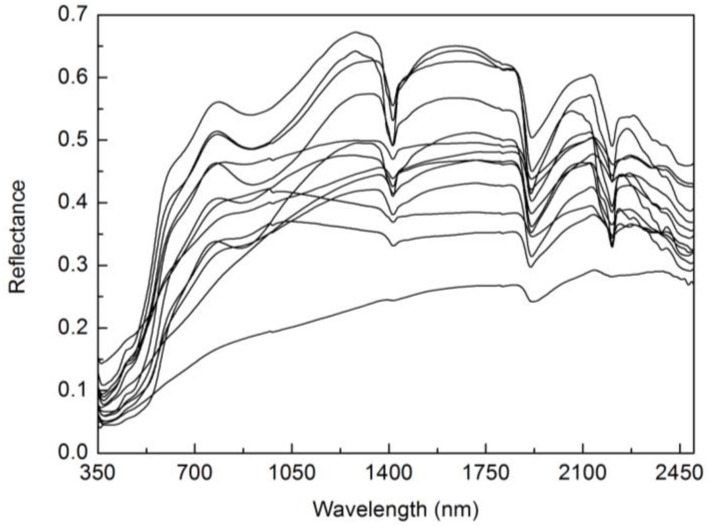
The 13 soil reflectances used to represent the possible range of spectral shapes for the PROSAIL model.

The underlying soil reflectances were also input variables for the PROSAIL model. The soil reflectances from a globally distributed soil spectral library released by the International Soil Reference and Information Centre (access at: http://www.isric.org), which contained various soil types with various properties and had representative of various soil types [19,43], were selected for the inputs of PROSAIL model. To remove data redundancy generated by similar soil reflectances and to avoid huge computations in PROSAIL simulations, several representative soil reflectances should be determined from the original data. The spectral angle mapper method [44,45] was used to evaluate the similarity of different soil reflectances and further determined the representative soil reflectances for PROSAIL model. The soil reflectances having spectral angle value smaller than 0.05 would be considered as similar soil reflectances which would be averaged as a representative soil reflectance. Finally, 13 soil reflectances were determined to represent the possible range of soil reflectances in the PROSAIL model (Figure 3).

It had been demonstrated that reasonable error of the input variables of radiative transfer model was permitted which did not lead to obvious loss of parameter inversion accuracy, therefore some input variables in the PROSAIL model could be fixed in the simulations [46,47]. Considering the objective of this study was to develop a general LAI estimation algorithm using GF-1 WFV data under various land surface conditions, the input variables of PROSAIL model were given reasonable ranges or fixed values (Table 3) based on previous studies, such as the Leaf Optical Properties Experiment 93 and the algorithms of CYCLOPES LAI product [22,26,48,49]. Therefore, the PROSAIL model could simulate the vegetation canopy reflectances based on the vegetation physicochemical parameters, geometric parameters and soil reflectances, meanwhile the remote sensing data could also obtain vegetation canopy reflectances by atmospheric correction, thus the remote sensing reflectance data were associated with LAI through the physical process of radiation transmission.

For any combination of the input variables, the canopy reflectance was computed for each wavelength from 0.4 to 2.5 μm at a spectral resolution of 1 nm, and then the canopy reflectance was resampled to simulate GF-1 WFV observations using the relative spectral response profiles. Because the relative spectral response profiles of the 4 WFV sensors on board GF-1 satellite were slightly different, the simulations of GF-1 WFV reflectance were separately generated for each WFV sensor. The canopy reflectance simulations using PROSAIL model resulted in 2,021,760 cases of matched reflectances and LAI values for each WFV sensor. To account for uncertainties in the satellite observations and model simulations, a white Gaussian noise with signal to noise ratio of 100 was added to the simulated reflectances, which were used as the learning dataset for the NNs.

### 4.2. Neural Networks

**Figure 4:**
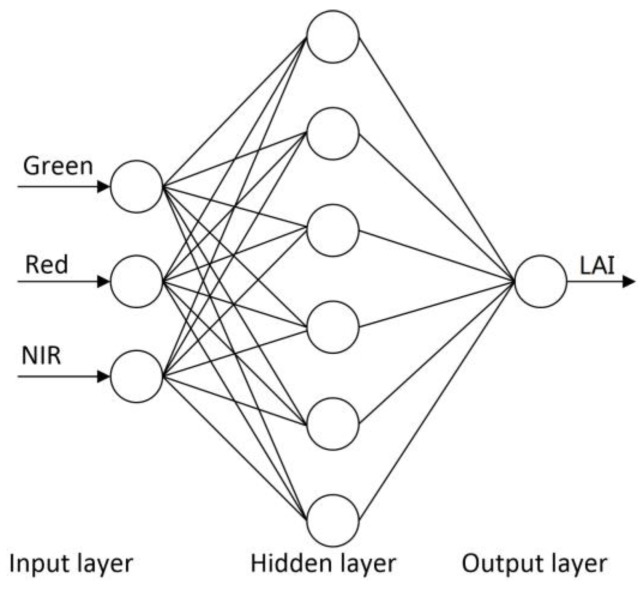
The architecture of the back propagation neural networks (BPNNs) used for LAI estimation from GF-1 WFV reflectance data.

The NNs mimic human learning to build relationships between variables, which can approximate multivariate non-linear relationships and are robust to noisy data [50,51], thus have been widely used for estimating land surface parameters from remote sensing data [7,52,53]. The popular back propagation NNs (BPNNs) were selected for LAI estimation algorithm development using GF-1 WFV data [4,7]. The BPNNs could learn from the training sample dataset and built relationships between reflectances and LAI, then the trained BPNNs could generate the optimal LAI estimates based on the actual reflectances from GF-1 WFV data. The architecture of the BPNNs used for LAI estimation from GF-1 WFV data was shown in Figure 4. The input variables of the BPNNs included the reflectances of the green, red and NIR bands, and the output variable was the corresponding LAI. The number of nodes in the hidden layer was set to six. The BPNNs activation functions in the hidden layer and output nodes were set to “sigmoid” and “linear”, respectively. The Levenberg–Marquardt minimization algorithm was used to calibrate the synaptic coefficients because of its efficient convergence capacity [19,54]. The training sample dataset made of pairs of reflectances and LAI, which were generated from the PROSAIL model simulations, was randomly split into two parts: 90% of the cases were used for training BPNNs, meanwhile the rest 10% of the cases were used to test the hyper-specialization during the training process. The BPNNs models were generated for each of the four GF-1 WFV sensors based on the specific training sample dataset for each WFV sensor.

### 4.3. LAI Estimating Procedure for GF-1 WFV Data

The NDVI generated from red and NIR band reflectances was an important indicator for vegetation growth conditions, and usually used to estimate LAI based on empirical relationships between LAI and NDVI [55,56,57]. Therefore, to avoid the presentation of abnormal NNs inversion values caused by the non-vegetation pixels, it was a good strategy to remove non-vegetation pixels using NDVI threshold value before LAI estimation using the NNs. In this study, the NDVI threshold value of 0.05 was used to distinguish vegetation and non-vegetation pixels. The pixels with NDVI value smaller than 0.05 was identified as non-vegetation pixels and LAI would be set to zero, whereas the LAI for other pixels would be estimated using the trained NNs, from which the GF-1 WFV data should be judged from that sensor and then adopt the corresponding trained NNs.

### 4.4. LAI Estimation Accuracy Assessment

Direct comparison of field LAI measurements with LAI estimates using GF-1 WFV land surface reflectance data is a reliable way to assess the accuracy of the proposed LAI estimation algorithm. Comparison between the field survey and predicted LAI will be performed by using five statistical indices: R^2^ of linear regression, root mean square error (RMSE), relative RMSE (RRMSE), Nash–Sutcliffe model efficiency coefficient (EF) [58] and coefficient of residual mass (CRM) [59]. The RMSE values show how much the predicted LAI values under- or over-estimate the field observations. The RRMSE is calculated by dividing the RMSE by the mean field observations. The EF value compares the predicted LAI values to the average value of the field observations. A negative EF value indicates that the average value of the field observations gives a better estimate than the predicted values. The CRM is a measure of the tendency of the developed LAI estimation method to overestimate or underestimate the field observations. A positive CRM value indicates that the method underestimates the field observations and the negative value indicates a tendency to overestimate. For a perfect fit between field observed and predicted LAI data, values of R^2^, RMSE, RRMSE, CRM, and EF should equal 1.0, 0.0, 0.0, 0.0 and 1.0, respectively.

## 5. Results

**Figure 5:**
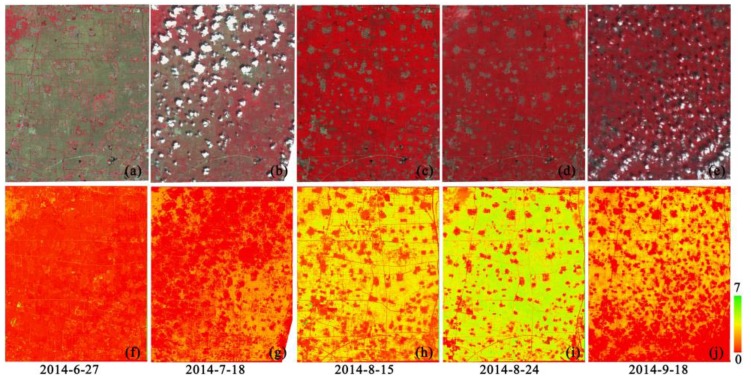
GF-1 WFV land surface reflectance data ((**a**) 27 June 2014, (**b**) 18 July 2014, (**c**) 15 August 2014, (**d**) 24 August 2014, and (**e**) 18 September 2014) and their corresponding LAI estimates ((**f**): 27 June 2014, (**g**) 18 July 2014, (**h**) 15 August 2014, (**i**) 24 August 2014, and (**j**) 18 September 2014).

The LAI estimation results using the proposed algorithm and the pre-processed GF-1 WFV land surface reflectance data are shown in Figure 5. In the visual aspect, the spatial and temporal distributions of LAI estimates are reasonable. As for the spatial distribution, LAI estimates using GF-1 WFV data acquired on 27 June generally show small values, because maize at this period is at the early emergence stage with small LAI, while larger LAI estimates only present at an orchard on the upper left corner of the study area. After July, with the rapid growth of maize, the large LAI estimates are mainly distributed in the area of farmland, and the LAI estimates at other areas are smaller. Meanwhile, LAI estimates in the cloud and cloud shadow regions are all zero. Regarding the temporal variations, LAI estimates of maize show low values in late June and continuous increases in July and mid-August, reaching the peak LAI values in the late August and a downward trend in mid-September. The temporal variation characteristics of LAI estimates exactly reflect the growth processes of maize from germination, rapid growth to peak value, and then gradually decline, which is in accord with the growth characteristics of maize. Therefore, the visual observation of the spatial and temporal characteristics of LAI estimates using GF-1 WFV data could preliminarily indicate the reasonability of the proposed LAI estimation algorithm.

**Figure 6:**
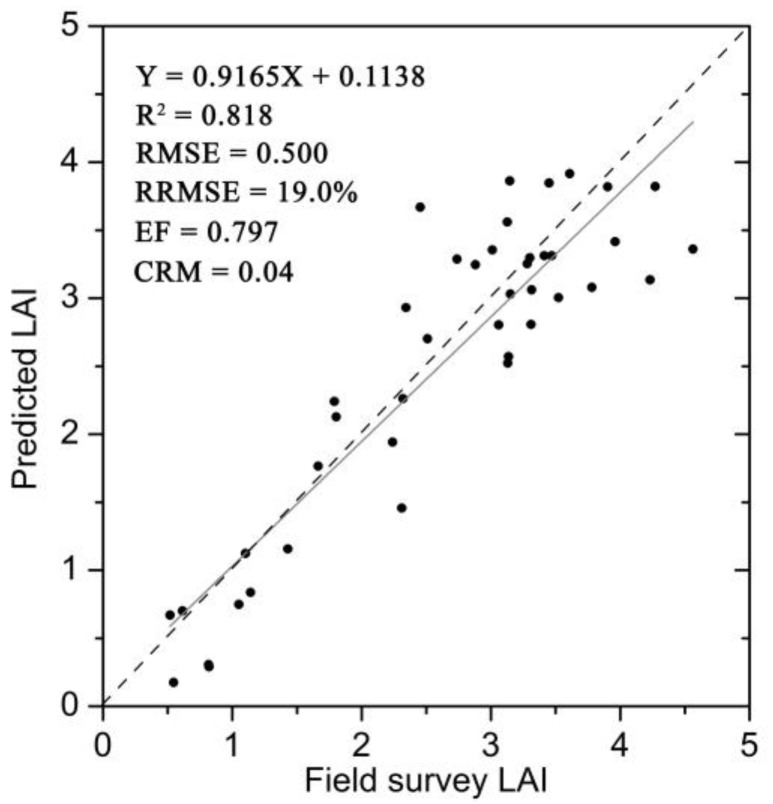
Scatter plots between field survey LAI and GF-1 WFV data predicated LAI.

The field LAI measurements located in the cloud and cloud shadow regions were deleted based on the visual observation, and the remaining field LAI measurements, which contained 43 LAI data, were used to directly validate the LAI estimates from GF-1 WFV data using the proposed algorithm (Figure 6). It could be seen that there was a good linear relationship between field LAI measurements and LAI estimates from GF-1 WFV data. The LAI estimation accuracy was satisfactory with values of R^2^, RMSE, RRMSE, CRM, and EF equaling 0.818%, 0.5%, 19.0%, 0.04% and 0.797%, respectively. In addition, some larger differences between the field survey LAI and the predicted LAI were observed in the region of large LAI values. The main reason might be that the field LAI values measured on 5 September were matched with LAI estimates interpolated from LAI estimates on 24 August and 18 September, whereas maize in this period was close to the mature stage with great LAI change rate, thus the interpolated LAI results might have some uncertainties. The other reason might be that the clouds influenced the atmospheric conditions of the study area which further influenced the accuracy of obtaining GF-1 WFV land surface reflectances and caused the uncertainties of LAI estimates.

In summary, the difference between the field LAI measurements and LAI estimates from GF-1 WFV data was small and the LAI estimation results were satisfactory. Therefore, the results indicated that the proposed LAI estimation algorithm for GF-1 WFV data was reliable and GF-1 WFV data could achieve acceptable performance on LAI estimation, which had the potential for providing high temporal and spatial resolution LAI dataset for related applications, such as agriculture monitoring. 

## 6. Discussion and Conclusions

This study proposed a LAI estimation algorithm for GF-1 WFV land surface reflectance data based on BPNNs with training samples generated from the radiative transfer model simulations under different soil and vegetation conditions, thus the algorithm could adapt to a variety of different underlying conditions. The validation results using field LAI measurements in an agriculture region with maize as the dominated crop type showed that the proposed algorithm could achieve satisfactory LAI estimation accuracy (e.g., RMSE = 0.50), which indicated GF-1 WFV data had good performance on LAI estimation and the algorithm had potential to operationally estimate LAI from GF-1 WFV land surface reflectances. The LAI estimation algorithm was automatically operated without prior knowledge on land cover, and no human interaction and no empirical model parameters were needed. Therefore, the proposed LAI estimation algorithm for GF-1 WFV data could overcome the difficulties in determination of model parameters for empirical LAI estimation methods, which were generally changed with time, region and vegetation types. In addition, the studies of LAI estimation using remote sensing data based on NNs trained over radiative transfer model simulations in the past mainly focused on the kilometric spatial resolution remote sensing data, and several global LAI products were generated from SPOT VEGETATION, MERIS and MODIS data. Therefore, the developed LAI estimation algorithm for decametric spatial resolution remote sensing data using NNs based on physical model was a new try in this study, and LAI with decametric spatial resolution would be more useful for agriculture, ecosystem and environment management than kilometric spatial resolution data which were usually larger than the typical scales of most landscapes.

There were also some limitations about this study, even though it achieved satisfactory LAI estimation performance. Firstly, the field LAI measurements for validation were collected from an agriculture region covering the main growing season of maize, though they had temporal–spatial representativeness of cropland and the agriculture monitoring was the main application field of LAI, exploratory investigations should be strengthened using much more validation data from various vegetation types in the future work. Moreover, the pre-processes of GF-1 WFV data were conducted manually. If an automated data preprocessing approach for radiation correction and geometric correction could be developed, a streamlined LAI estimation workflow would be formed from the original DN values to LAI estimates, which would make the LAI estimation from GF-1 WFV data much simpler and quicker. Finally, the accuracy assessment of LAI estimation algorithm was difficult to achieve due to the difficulties in obtaining real LAI values from field measurements to match with the pixel level LAI estimates. Currently, using plant canopy analyzer to obtain field LAI measurements was the commonly used method, which could obtain relatively accurate LAI data. However, some errors might be made when matching the field LAI measurements and pixel scale LAI estimates due to the non-absolute uniformity of the land surface, though averaging the multi-point measurements could reduce this error to a certain extent. Perhaps using an unmanned aircraft system flying in the lower altitude to take photos from the nadir and developing corresponding LAI extraction algorithm from photos was a considerable way for real LAI data collection. Although the field LAI measurements had many uncertainties, they still played an important role in assessing LAI estimation algorithms using remote sensing data, and more effective real LAI obtaining approaches would be expected in the future.

In conclusion, the proposed LAI estimation algorithm for GF-1 WFV data was reliable and GF-1 WFV data were confirmed as having a good performance on LAI estimation, which could provide reliable high spatial and temporal resolution LAI data for related applications. Further work would focus on validating the proposed LAI estimation algorithm using field LAI measurements with less uncertainties under various land cover type conditions.

## Figures and Tables

**Table 1:** The main characteristics of GF-1 wide field view (WFV) data used in this study.

WFV Sensor	Date (dd/mm/yy)	Matched Field Survey Date	Data Quality
WFV1	27 June 2014	27 June 2014	Good
WFV2	18 July 2014	21 July 2014	With cloud
WFV1	15 August 2014	14 August 2014	Good
WFV3	24 August 2014	5 September 2014	Good
WFV4	18 September 2014	5 September 2014	With cloud

**Table 2:** The calibration coefficients of GF-1 WFV data in 2014.

WFV Sensor	Bands	Gain	Offset
WFV1	Blue band (Band1)	0.2004	0
	Green band (Band2)	0.1648	0
	Red band (Band3)	0.1243	0
	NIR band (Band4)	0.1563	0
WFV2	Blue band (Band1)	0.1733	0
	Green band (Band2)	0.1383	0
	Red band (Band3)	0.1122	0
	NIR band (Band4)	0.1391	0
WFV3	Blue band (Band1)	0.1745	0
	Green band (Band2)	0.1514	0
	Red band (Band3)	0.1257	0
	NIR band (Band4)	0.1462	0
WFV4	Blue band (Band1)	0.1713	0
	Green band (Band2)	0.1600	0
	Red band (Band3)	0.1497	0
	NIR band (Band4)	0.1435	0

**Table 3:** The input variables of PROSAIL model for LAI estimation algorithm development.

Parameters	Units	Value Range	Step
LAI	m^2^/m^2^	0–7	0.2
ALA	°	30–70	10
N	-	1–2	0.5
C_ab_	μg/cm^2^	30–60	10
C_m_	g/cm^2^	0.005–0.015	0.005
C_ar_	μg/cm^2^	0	-
C_w_	cm	0.005–0.015	0.005
C_brown_	-	0–0.5	0.5
Hot	-	0.1	-
Solar zenith angle	°	25–55	10
